# Severe primary hypothyroidism in an apparently asymptomatic 19-year-old woman: a case report

**DOI:** 10.1186/s13256-021-02677-w

**Published:** 2021-03-03

**Authors:** Rania Dannan, Sulaiman Hajji, Khaled Aljenaee

**Affiliations:** 1 Kuwaiti Board of Internal Medicine, Ahmadi, Kuwait; 2 Al-Adan Hospital, Kuwait City, Kuwait

**Keywords:** Hypothyroidism, TSH, Asymptomatic, Screening, Case report

## Abstract

**Background:**

Hypothyroidism is diagnosed on the basis of laboratory tests because of the lack of specificity of the typical clinical manifestations. There is conflicting evidence on screening for hypothyroidism.

**Case presentation:**

We report a case of an apparently healthy 19-year-old Kuwaiti woman referred to our clinic with an incidental finding of extremely high thyroid-stimulating hormone (TSH), tested at the patient’s insistence as she had a strong family history of hypothyroidism. Despite no stated complaints, the patient presented typical symptoms and signs of hypothyroidism on evaluation. Thyroid function testing was repeated by using different assays, with similar results; ultrasound imaging of the thyroid showed a typical picture of thyroiditis. Treatment with levothyroxine alleviated symptoms and the patient later became biochemically euthyroid on treatment.

**Conclusion:**

There is controversy regarding screening asymptomatic individuals for hypothyroidism; therefore, it is important to maintain a high index of suspicion when presented with mild signs and symptoms of hypothyroidism especially with certain ethnic groups, as they may be free of the classical symptoms of disease.

## Background

We report a case of an apparently healthy 19-year-old Kuwaiti woman referred to our clinic with an incidental finding of extremely high thyroid-stimulating hormone (TSH), tested at the patient’s insistence as she had a strong family history of hypothyroidism. Despite no stated complaints, the patient presented typical symptoms and signs of hypothyroidism on evaluation. This raised the question of who should be screened for hypothyroidism. Screening for hypothyroidism refers to the measurement of thyroid function in asymptomatic populations who are at high risk of having thyroid disease, or patients who have mild, nonspecific symptoms, such as tiredness. Here, we summarize the conflicting evidence regarding screening for hypothyroidism, and emphasize the importance of maintaining a high index of suspicion for hypothyroidism even when presented with mild signs and symptoms.

## Case report

A previously healthy, seemingly asymptomatic 19-year-old woman was referred to us by her general practitioner because of an extremely high TSH level of 1099 mlU/L, detected on random testing at the patient’s request because of her family’s history of autoimmune thyroid disease. The patient reported fatigue with excessive sleepiness lasting more than 14 hours per day, depressed mood, inexplicable weight gain, decreased appetite, hair loss, constipation, and menorrhagia, all indicative of a hypothyroid state. There was no history of chronic medical conditions or regular medication use, although the patient reported the use of over-the-counter paracetamol for headaches and dysmenorrhea. Physical examination showed normal vital parameters (weight 72 kg; height 160 cm; blood pressure 124/80 mmHg; pulse 56 bpm) and classical signs of hypothyroidism: periorbital puffiness and loss of outer third of her eyebrows and dry skin that was not coarse. Neck examination showed no scars and upward thyroid movement with deglutition, a smooth palpable goiter without nodules, and no palpable lymph nodes. Lower limb tone and power were intact despite slow relaxation of ankle reflexes.

**Fig. 1:**
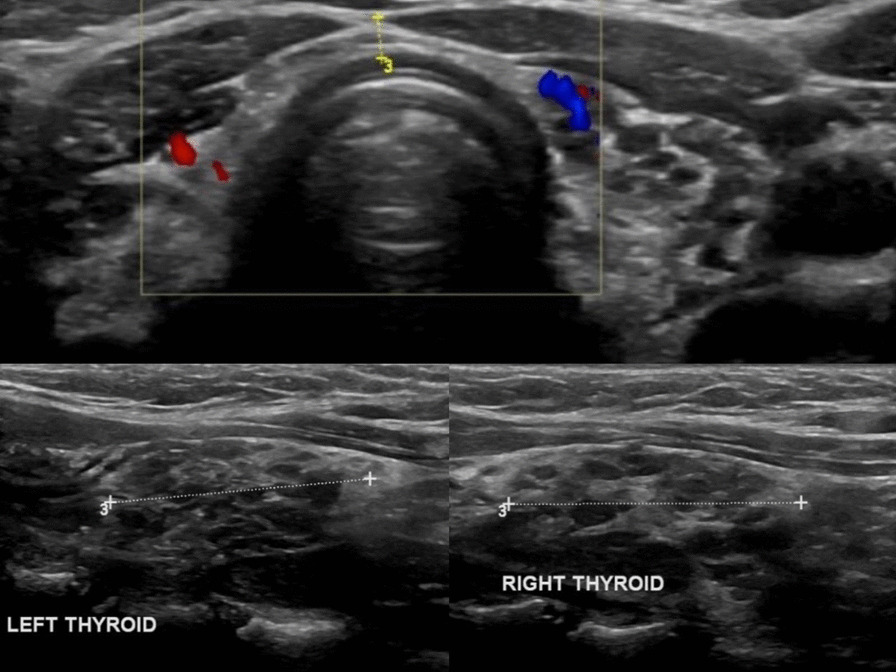
Ultrasound of the thyroid showing diffuse cystic changes replacing much of the normal thyroid tissue with a “Swiss cheese” appearance

Laboratory findings from before and after treatment initiation are presented in Table 1. At the baseline in April 2019, the patient’s TSH was 1099 mlU/L (Roche assay) and free thyroxin (T4) was 0.7 pmol/L; the TSH level was retested in a Siemens assay, with a similar result (TSH 991 mlU/L). A subsequent anti-thyroid peroxidase (anti-TPO) antibodies test showed high levels (42 lU/mL). Complete blood count, renal function, liver enzymes, and lipid profile were tested; the hemoglobin level was 10 g/dL, with a microcytic hypochromic picture, most likely due to iron-deficiency anemia because the ferritin level was 6 ng/mL. The lipid profile showed dyslipidemia (total cholesterol 6.50 mmol/L, low-density lipoprotein 4.09 mmol/L, triglycerides 1.56 mmol/L). Renal function was normal. Ultrasound imaging of the thyroid showed diffuse cystic changes replacing much of the normal thyroid tissue with a “Swiss cheese” appearance (Fig. 1). The patient was started on levothyroxine in April 2019, and subsequent TSH and free T4 tested in December 2019 were 2.320 mIU/L and 17.5 pmol/L, respectively; symptom resolution was noted after a few weeks on levothyroxine (100 µg) supplementation, and the patient became biochemically euthyroid after 6 months.

**Table 1:** Laboratory results

	Reference ranges	April 2019	December 2019 (on levothyroxine)
TSH (mIU/L) (Roche assay)	0.27–4.2	1099	2.320
TSH (mIU/L) (Siemens assay)	0.27–4.2	991	2.320
Free T4 (pmol/L)	12–22	0.7	17.5
Anti-TPO antibodies (lU/mL)	5–34	42.35	**–**
Hb (g/dL)	120–140	10.5	**–**
MCV (fL)	78–90	79.5	**–**
Ferritin (ng/mL)	10–120	6	**–**
Total cholesterol (mmol/L)	3.1–5.1	6.50	**–**
LDL cholesterol (mmol/L)	1.5–3.84	4.09	**–**
HDL cholesterol, (mmol/L)	1.04–1.9	1.69	**–**

*TSH* thyroid-stimulating hormone, *T4* thyroxin, *Anti-TPO* anti-thyroid peroxidase, *Hb* hemoglobin, *MCV* mean corpuscular volume, *LDL* low-density lipoprotein, *HDL* high-density lipoprotein

## Discussion

Hypothyroidism is a disorder of thyroid hormone deficiency that affects 0.2–5.3% of Europeans and 0.3–3.7% of Americans [1]. Clinical manifestations vary by patient age as well as the duration and severity of thyroid hormone deficiency. Therefore, a definite diagnosis is primarily made on the basis of biochemical testing [2]. There is an ongoing debate about the optimal normal ranges of TSH and T4. A TSH level higher than 4.2–4.5 mlU/L and a free T4 less than 10 pmol/L confirms hypothyroidism [3]. Therapy aims to supplement thyroxine to alleviate signs and symptoms of hypothyroidism, and to normalize serum thyrotropin without overtreatment [2].

In younger populations, typical symptoms of hypothyroidism are usually present and can facilitate diagnosis, whereas a diagnosis in the elderly is difficult because of seemingly asymptomatic presentation and thus necessitates a high index of suspicion in clinicians to predict hypothyroidism [2]. Moreover, symptoms and signs of hypothyroidism vary from person to person depending on age, gender, and origin [4–6]. Table 2 presents the commonest symptoms and signs of patients with hypothyroidism in Saudi Arabia, Oman, and Australia; in all three countries, tiredness, which was documented in 56%, 25%, and 84% of patients, respectively, was the most common presenting symptom [4–6]. Our patient presented with symptom unawareness despite an extremely high TSH level incidentally detected on biochemical testing, but had typical symptoms of hypothyroidism on clinical evaluation. This raises the question of hypothyroidism screening: who should be screened? And when should they be screened?

**Table 2:** Frequency (%) of symptoms in patients with hypothyroidism

	Saudi Arabia [4]	Oman [4]	Australia [5]
Symptoms			
Tiredness	56	25	84
Cold intolerance	38	–	59
Constipation	36	20	40
Weight gain	36	10	38
Menstrual disturbances	36	3	58
Hair loss	10	–	44
Decreased sweating	1	–	34
Hoarseness	31	–	55
Sleepiness	14	–	40
Coarse hair	11	–	40
Dry skin	35	10	–
Depression	11	–	–
Signs			
Coarse skin	53	–	72
Delayed reflex relaxation	32	–	–
Goiter	24	10	21
Periorbital puffiness	17	–	40
Slow movements	11	–	44
Bradycardia	6	–	–
Slow speech	5	–	48
Lower limb edema	3	3	–

Screening for hypothyroidism refers to the measurement of thyroid function in asymptomatic populations who are at high risk of having thyroid disease, or patients who have mild, nonspecific symptoms, such as tiredness. Screening is carried out by measuring serum TSH levels [6], but there are conflicting recommendations on screening. The American Thyroid Association (ATA) recommends screening for hypothyroidism in all adults 35 years and older every 5 years and in certain high-risk individuals [6]. However, the US Preventative Task Force (USPTF) found insufficient evidence for screening of thyroid dysfunction and emphasizes the uncertainties surrounding potential clinical benefits [7]. Similarly, the Royal College of Physicians in London found no justification for screening for hypothyroidism even in the elderly and individuals with a strong family history of thyroid disease [8]. Exceptions include screening of newborn babies for congenital hypothyroidism and patients with previous thyroid surgery or radioactive iodine treatment as well as patients receiving long-term lithium or amiodarone therapy [8]. There are no strong recommendations for screening of the non-pregnant asymptomatic population for hypothyroidism. Therefore, many asymptomatic patients with overt hypothyroidism could remain undiagnosed and untreated.

## Conclusion

Our 19-year-old patient presented to the clinic with no complaints, but further assessment revealed typical signs and symptoms of hypothyroidism and a TSH level higher than 1000 mlU/L. All symptomatic patients should be evaluated for hypothyroidism, but screening of asymptomatic individuals is controversial and not recommended by certain organizations. This report is presented to recommend a lowering of the screening threshold for hypothyroidism and to motivate physicians to boost their index of suspicion to diagnose the disease, especially with certain ethnic groups, as they may be free of the classical symptoms so severe hypothyroidism can be missed.

## Data Availability

Data sharing not applicable to this article as no datasets were generated or analyzed during the current study.

## Abbreviations

Anti-TPO: Anti-thyroid peroxidase
ATA: American Thyroid Association
Hb: Hemoglobin
HDL: High-density lipoprotein
LDL: Low-density lipoprotein
MCV: Mean corpuscular volume
T4: Thyroxin
TSH: Thyroid-stimulating hormone
USPTF: The United States Preventive Services Task Force
